# Structural Evolution of Manganese Prussian Blue Analogue in Aqueous ZnSO_4_ Electrolyte

**DOI:** 10.1002/smll.202404584

**Published:** 2024-08-06

**Authors:** Min Li, Mariam Maisuradze, Angelo Mullaliu, Ilaria Carlomagno, Giuliana Aquilanti, Jasper Rikkert Plaisier, Marco Giorgetti

**Affiliations:** ^1^ Department of Industrial Chemistry University of Bologna Campus Navile Via Piero Gobetti 85 Bologna 40139 Italy; ^2^ Department of Chemistry KU Leuven Leuven 3001 Belgium; ^3^ Elettra – Sincrotrone Trieste s.s. 14, km 163.5 Trieste 34149 Italy

**Keywords:** aqueous Zn‐ion battery, manganese hexacyanoferrate, structural evolution, synchrotron X‐ray techniques

## Abstract

Among different Prussian Blue Analogues (PBAs), manganese hexacyanoferrate (MnHCF), with open framework and two abundant electroactive metal sites, exhibits high potential for the grid‐scale aqueous rechargeable zinc‐ion batteries (ARZIBs) application. Until now, the intercalation mechanism of Zn^2+^ into MnHCF has not been clearly illustrated. In this work, combining different synchrotron X‐ray techniques, the structural and microscopic evolution of MnHCF in 3 m ZnSO_4_ electrolyte is comprehensively studied, and a thorough understanding of the intercalation/release dynamic, in terms of local and long‐range domain, is provided. The elemental distribution and structural information of Fe, Mn, Zn inside MnHCF electrodes is obtained from the X‐ray fluorescence (XRF) elemental maps and X‐ray absorption spectroscopy (XAS). The in‐depth analysis of extended X‐ray absorption fine structure (EXAFS) signals confirm that the rearrangement of Mn site, evidencing the cleavage of the Mn*─*N bond with the formation of a Mn*─*O bond, in an octahedral environment. The phase transformation of MnHCF takes place exclusively during the 1st cycle, and a mixture of rhombohedral and cubic zinc hexacynoferrate (ZnHCF) phases are formed during the first charge process. Thereafter, the newly formed cubic ZnHCF phase becomes the only stable one, existing in the subsequent cycles and exhibiting excellent electrochemical stability.

## Graphical abstract

Combining multiple synchrotron X‐ray techniques, the local and long‐range structural and electronic modification of manganese hexacyanoferrate in aqueous rechargeable zinc‐ion batteries are comprehensively studied. The local metal configuration and crystal structure evolution are closely related to the electrochemical performance, which provides a thorough understanding of the intercalation/release dynamic, and structure–property–performance relationships.

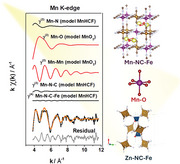

## 1 Introduction

Aqueous rechargeable zinc‐ion batteries (ARZIBs) have attracted extensive attention as one of the most promising postlithium ion battery candidates for the large‐scale electrochemical energy storage, because of their low cost, intrinsic safety, and environmental friendliness.^[^ ^1^, ^2^ ^]^ The ARZIBs usually consists of a Zn^2+^ storage cathode, Zn metal anode, and aqueous Zn^2+^ containing electrolyte. Comparing with other metal anodes, the benefit of Zn is that it can be used directly in aqueous electrolyte, and also exhibits high theoretical gravimetric and volumetric capacity of 820 mAh g^−1^ and 5855 mAh cm^−3^, respectively, with low electrochemical potential (−0.76 V vs SHE).^[^ ^3^, ^4^ ^]^ Manganese hexacyanoferrate (MnHCF), as one of the Prussian Blue Analogues (PBAs), has attracted wide attention as a promising cathode material for both Li‐ion and post‐Li ion batteries.^[^ ^5^, ^6^, ^7^, ^8^, ^9^, ^10^ ^]^ The application of MnHCF in aqueous Zn‐ion batteries has been first reported by Hou et al.^[^ ^11^ ^]^ by using Na_2_MnFe(CN)_6_ nanotubes as the cathode and a zinc metal sheet as the anode, which exhibited a high initial specific capacity of 140 mAh g^−1^ in Na_2_SO_4_/ ZnSO_4_/ sodium dodecyl sulfate (SDS) electrolyte, with 75% capacity retention after 2000 cycles. However, MnHCF normally suffers from fast capacity fading problem in aqueous Zn^2+^ electrolyte, due to the severe dissolution of Mn and irreversible intercalation of Zn^2+^.^[^ ^12^, ^13^, ^14^, ^15^ ^]^ In addition to that, the Jahn–Teller distortion of Mn^3+^ during charge/discharge processes would aggravate the structural deformation and deteriorate the electrochemical performance. Compared to the extensive mechanism study of manganese oxide ARZIBs,^[^ ^16^, ^17^, ^18^, ^19^, ^20^, ^21^, ^22^, ^23^ ^]^ the research on the application and the mechanism of MnHCF in ARZIBs is quite limited.

To figure out what exactly happened to the electrode material during the charge/discharge process in ARZIBs, e.g., where Zn^2+^ resides within the MnHCF framework during cycling, and how does its crystal structure change, our group have employed ex situ XAS technique to record Mn, Fe, and Zn K‐edge spectra at different charge and discharge states.^[^ ^24^ ^]^ We found a ‐Zn‐NC‐Fe‐ structure formed in all cycled electrodes in 3 m ZnSO_4_ electrolyte, which indicates that there is a replacement of Mn by Zn during the charge/discharge process, and this also partly explained the decrease of capacity of MnHCF during the cycling, due to the chemical inertness of Zn.^[^ ^25^, ^26^ ^]^ Similar results were also reported by Cao et al.,^[^ ^13^ ^]^ Deng et al.,^[^ ^14^ ^]^ and Ni et al.^[^ ^15^ ^]^ A phase transformation from MnHCF to ZnHCF (Zn_3_[Fe(CN)_6_]_2_) phase was observed in different aqueous Zn^2+^ electrolytes, e.g., 1 m ZnSO_4_ +0.1 m MnSO_4_,^[^ ^13^ ^]^ 30 m KFSI +1 m Zn(CF_3_SO_3_)_2_,^[^ ^14^ ^]^ and 3 m ZnSO_4_ or 3 m Zn(CF_3_SO_3_)_2_.^[^ ^15^ ^]^ During the phase transformation process, it is widely reported that the Mn experience severe dissolution problem, while the study about the detailed local configuration change of Mn inside MnHCF structure is rare and controversial. Deng et al.^[^ ^14^ ^]^ mentioned that with the intercalation of Zn^2+^, the octahedral MnN_6_ became highly distorted and even turned into tetrahedral MnN_4_, with the content of Mn decreased gradually and dropped to near zero after 100 cycles. Ni et al.^[^ ^15^ ^]^ found that while the KMHCF transforms into the rhombohedral Zn_3_[Fe(CN)_6_]_2_ during the first charge process, it is also accompanied by the Mn ions dissolution and redeposition on the electrode as manganese oxides.

Thus, to comprehensively understand the intercalation process of Zn^2+^ in MnHCF electrode, we built an ARZIB system by using aqueous ZnSO_4_ (3 m) electrolyte, which is the very commonly used and cheap electrolyte for ARZIBs. The detailed structure and composition evolution of MnHCF in ZnSO_4_ electrolyte was studied using operando and ex situ synchrotron X‐ray techniques, including XRD, XAS, and XRF. The elemental distribution of the electrodes was collected by the synchrotron radiation XRF technique. Quantitative XRF results show that the Zn content increased rapidly during the 1st charge process and reached stable and homogeneous distribution during the subsequent cycles. On the contrary, the Mn content decreased a lot after 1st discharge, and then increased and became evenly distributed. XAS at both Mn and Fe K‐edge indicate that most of the local structural rearrangement takes place at the Mn site, evidencing the cleavage of the Mn*─*N bond with the formation of a Mn*─*O bond, yet in an octahedral environment. This piece of information, statistically evident in samples after 10 cycles. Combing the linear combination fitting (LCF) of X‐ray absorption near‐edge structure (XANES) spectra, the formation of MnO_2_ as a main manganese‐ “survival” component in the cathode was proposed. While the XRD results indicate that the newly formed MnO_2_ only exhibits a local octahedral configuration, without a long‐range ordered crystal structure. The phase transformation of MnHCF was confirmed by XRD results, and a mixture of rhombohedral and cubic ZnHCF phases were formed during the first charge process. After that, the new cubic ZnHCF phase became the only stable phase participating in the subsequent cycling, and it exhibited high cycling stability.

## 2 Results and Discussion

### 2.1 The Modification of Fe, Mn, and Zn Sites Inside MnHCF

**Figure 1:**
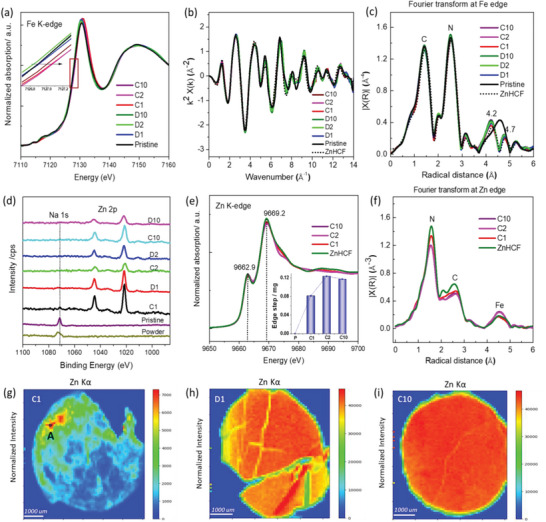
a) Ex situ XANES of Fe K‐edge at different charge/discharge states (pristine electrode, first charge/discharge (C1/ D1), second charge/discharge (C2/ D2), and 10th charge/discharge (C10/ D10)). b) *k* ^2^‐weighted EXAFS signals, as well as the c) corresponding Fourier transforms (FTs) signal. d) Ex situ Na 1s and Zn 2p XPS spectra at different charge/discharge states, compared with MnHCF powder and pristine electrode samples. e) Ex situ XANES Zn K‐edge data of C1, C2, C10, and ZnHCF powder sample, inset is the XANES edge step value of all charged electrodes. f) corresponding FTs of C1, C2, C10, and ZnHCF powder electrodes *k* ^2^‐weighted EXAFS signal. g–i) Intensity distribution of the Zn Kα fluorescence maps for C1, D1, and C10 electrodes, the irregular shape and cracks are due to the breakage of electrodes during the collection and sample preparation process.

XAS is an element specific technique and represents an ideal method to monitor the local structural changes of metal‐sites in different charged and discharged electrodes. As observed in **Figure** **1**a, all the cycled electrodes exhibit the same Fe K‐edge XAS spectral shape as the pristine one. A minor difference is observed in a small energy shift for all the charged electrodes, this is due to the increased oxidation state and is consistent with our previous report.^[^ ^24^ ^]^ A significant modification was observed at the third FT peak of the *k* ^2^‐weighted EXAFS signal (Figure 1c), which is due to the Mn, according to the framework structure of MnHCF (‐Fe‐C‐N‐Mn‐). For all the cycled electrodes, the third shell was changed from a broad peak (≈4.6 Å) to a doublet having a relatively tall and a small feature at around 4.2 and 4.7 Å, respectively. Noteworthy, the FTs of Fe K‐edge of the ZnHCF standard exhibit a similar signal at this distance (around 4.2 Å). It means that the Mn sites might be replaced by Zn, which is characterized by a shorter Zn*─*N bond distance with respect to Mn‐N. It is worth noticing that the first two peaks of the FT Fe K‐edge of the MnHCF series are superimposable, once again testifying that the ‐Fe‐C‐N‐ structural unit of the ‐Fe‐C‐N‐Mn (Zn)‐ framework is retained, and no major structural changes are taking place.

With the release of Na^+^ ions from MnHCF structure during the first charge process, simultaneously the content of Zn was evidently increased. It was detected in all cycled electrodes, even in the charged ones, as the XPS data demonstrated in Figure 1d. Therefore, the Zn K‐edge XANES and the corresponding FTs of EXAFS data of the cycled electrodes were collected and compared with the Zn K‐edge data of ZnHCF powder, as displayed in Figure 1e,f; indeed, the same spectral feature was observed for all the Zn K‐edge data. The inset edge step value (Figure 1e), which is proportional to the Zn content inside the electrodes, indicates that certain amount of Zn is clearly visible in C1, after increasing even more and relatively stabilizing in C2 and C10.

The content and distribution of Zn inside the electrodes were analyzed by synchrotron radiation XRF, as shown in Figure 1g–i. The Zn distribution was evaluated from the intensity distribution of the Zn Kα line in the electrodes C1, D1, and C10. The content, on the other hand, was assessed through the fitting of the cumulative XRF spectra (shown in Figure S1, Supporting Information). To reliably compare the Zn (and Mn) content across the different samples, we calculated the molar ratio Zn/Fe (and Mn/Fe). Thanks to the relatively stable content of Fe, which provides a reliable term of comparison to observe elemental trends among the samples. Our data show that the Zn/Fe ratio increased dramatically from 0.098 (C1 electrode) to 3.16 (D1 electrode) and remained at 2.78 in C10 (Figure S1d, Supporting Information), which also reflected on the Zn Kα fluorescence image (Figure 1g–i). The distribution of Zn became quite homogeneous inside D1 and C10 electrodes, compared to C1 electrode. Inside the C1 electrode, a hot spot was chosen at the edge part with high Zn content (Figure 1g, A spot), and the corresponding XANES spectra of Zn K‐edge were collected (Figure S2, Supporting Information). The XANES spectrum exhibits the same Zn K‐edge spectral features as C1 electrode and ZnHCF powder sample, which indicates that the Zn local configuration at A spot is the same. In addition, this observation excludes the possibility of having an adsorbed Zn^2+^, for instance, on the surface of the material.

**Figure 2:**
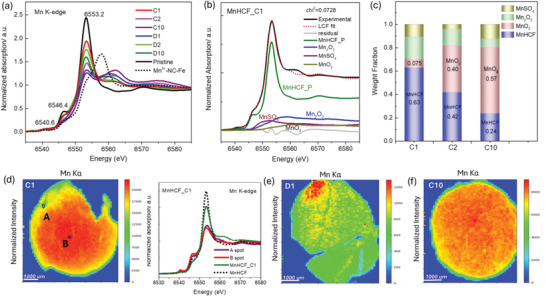
a) XANES spectra of Mn K‐edge at all cycled states (pristine electrode, C1, D1, C2, D2, C10, and D10 states). b) LCF of C1 XANES data with MnHCF, MnO_2_, Mn_2_O_3_, and MnSO_4_ Mn K‐edge data. c) LCF results of all charged electrodes. Intensity distribution of the Mn Kα fluorescence maps for d) C1 electrode, and XANES spectra at Mn K‐edge collected at A and B spots, compared with Mn K‐edge data of C1 and MnHCF pristine electrodes, as well as in e) D1 electrode and f) C10 electrode.

To confirm the large modification of the Mn local site during the insertion of Zn^2+^, the relative Mn K‐edge XANES data are displayed in **Figure** **2**a. The pre‐edge peak at around 6540.6 eV is attributed to the electronic transition 1s to 3d t_2g_/e_g_ orbitals, and the one at 6546.4 eV to the 1s to 4p or metal‐to‐ligand charge‐transfer (MLCT).^[^ ^27^, ^28^ ^]^ The edge region, which is the rising portion of the XANES spectra, is closely related the oxidation state of the metal sites. Compared to Mn^III^HCF (Figure 2a), the energy shift and spectral shape of C1, C2, and C10 electrodes look totally different, which indicates that the Mn inside these electrodes differ from the characteristic of Mn^3+^‐NC‐Fe framework. Another obvious difference was observed in the edge resonance region and in the main peak (white line) intensity at around 6553.2 eV, which was evidently decreased in all the cycled electrodes, with respect to the pristine MnHCF electrode. Normally, the intensity of the white line is influenced by the partial density of the unoccupied electronic states and increases when they have higher value near the absorption edge.^[^ ^29^ ^]^ Therefore, assuming the Mn^2+^ is oxidized to Mn^3+^, which conveys the increase of the empty d‐orbital contribution, a white line of high intensity is expected, which is not the case here. Besides that, the white line intensity is also affected by the chemical environment of the absorbing atom. Different chemical species or local coordination can lead to variations in the pre‐edge region intensity.^[^ ^28^, ^30^ ^]^ Thus, the reason behind the aforementioned decrease might be not only due to the change of electronic structure of the absorbing atom, but also its local atomic environment. To figure out the plausible existing state of Mn inside the cycled electrode, a linear combination fitting of Mn K‐edge XANES data with different Mn‐based standard compounds was conducted, as shown in Figure 2b. Although the quantification of the several species in XANES analysis is a quite difficult task, the contribution from Mn_2_O_3_, MnSO_4_, and MnO_2_ species is evidently observed here. In particular, the contribution of the MnO_2_ increased from 0.075 in C1 to 0.57 in C10 state (Figure 2c).

The XRF maps showing the Mn distribution in C1, D1, and C10 electrodes, as reported in Figure 2d–f. For the C1 electrode, the Mn is still quite homogeneously distributed, compared to D1 electrode, where the severe dissolution and replacement occurred on Mn site. An evenly distributed Mn was observed in C10 electrode, and the Mn/Fe ratio increased from 0.088 in D1 to 0.56 in C10 (Figure S1d, Supporting Information), which can be explained by the redeposition of Mn, as reported by Ni et al.^[^ ^15^ ^]^ Two hot spots were chosen inside C1 electrode: one is at the edge part with low Mn content (A spot), the other is in the center (B spot). The XANES spectra of A and B spots exhibit similar Mn K‐edge spectra, with an evident decrease in the white line, compared to the pristine MnHCF electrode Mn K‐edge data. To further confirm the existing state of Mn inside the cycled electrode, a more detailed EXAFS analysis is required.

**Figure 3:**
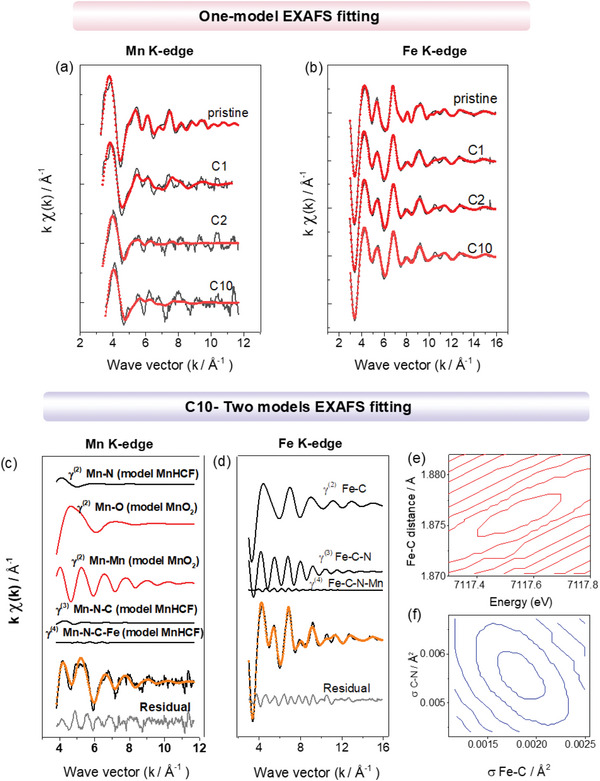
One model EXAFS fitting analysis of a) Mn K‐edge and b) Fe K‐edge signals of pristine, C1, C2, and C10 electrodes, comparison of experimental (—) and theoretical (‐∙‐) *k* ^2^‐ extracted signals. Details of the two models EXAFS analysis of the c) Mn K‐edge and d) Fe K‐edge signals of C10 electrode. Each panel of the figure shows the individual EXAFS contributions, in terms of two‐body, three‐body, and four‐body signals, to the total theoretical signal. e,f) Two‐section plots (contour plots) for the error parameters determination. The inner elliptical contour corresponds to 95% confidence level.

Thus, to gain a deep understanding of the local structure of Mn site, the EXAFS analysis at Mn K‐edge was conducted. **Figure** **3**a,b displays the EXAFS fitting results obtained at the Mn and Fe K‐edges, respectively, for the pristine, C1, C2, and C10 electrodes. Spectra have been fitted with one model, based on the pristine structure of the MnHCF.^[^ ^31^ ^]^ Evidently, this model well applies on the pristine sample (at both Fe and Mn local structures) but fails at Mn K‐edge EXAFS for C1, C2, and C10. On the other hand, the model still fits the EXAFS signals at the Fe K‐edge. These experimental findings indicate that the local structure of Fe site is not modified in various samples, and the network of octahedral Fe (CN)_6_ structural units still hold in all samples. This is not surprising, based on our previous report.^[^ ^24^ ^]^ and above discussion: Mn‐sites experienced the dissolution and replacement by Zn^2+^ during cycling, and therefore the Fe‐C‐N‐ (Mn or Zn) local structural arrangements is retained, regardless the identity of the transition metal linked to the N‐ end of the ‐NC‐ ligand.

On contrary, the Mn local atomic site requires a particular attention. The LCF XANES above suggested the occurrence of MnO_2_ phase in addition to the standard MnHCF phase. Indeed, the FTs of the EXAFS signals at the Mn K‐edge for samples C1, C2, and C10 also indicate a strong first shell modification for the Mn site (Figure S3, Supporting Information). As EXAFS is sensitive to the mean local structural environment in case of bi‐ or multicomponent systems, the EXAFS analysis at the Mn K‐edge would be very challenging, but still possible. By following the strategy available in the literature for the Au L3 EXAFS case,^[^ ^32^ ^]^ we have adopted a similar approach, and conducted the 2‐models fitting to the EXAFS signal for the C10 sample, as it is displayed in Figure 3c,d; and Figure S4 (Supporting Information). It reports the details of the 2‐models EXAFS analysis for sample C10, in terms of individual single contribution to the theoretical ones. The two chosen models were MnHCF and MnO_2_. The agreement of the theoretical signal to the experimental one is striking, and far better than the one model fitting for sample C10. The fit outcomes indicated that the total EXAFS experimental signal is reproduced using an estimation (in normalized unity) of 0.20(5) and 0.62(8) for the MnHCF and MnO_2_ models, respectively. It is remarkable that the quantification is comparable to the one obtained from the XANES LCF analysis. To the side of the observed bond lengths for manganese, the outcome is the following: Mn coordinates 6 oxygen at 1.816(5) Å, forming a MnO_6_ figure for the MnO_2_ local structure (this account around for the 60% of the Mn), and an additional Mn‐N, NC, and Fe‐C of the Mn‐N‐C‐Fe structural network for another 20%, ascribable to the hexacyanoferrate network, at 2.10(3), 1.172(3), and 1.876(3) Å (Table S1, Supporting Information), respectively. The improved quality of the fits respect to the one‐model is striking, thus underlying the reliability of the present data analysis, which also confirmed from the experimental errors given by the contour plots (Figure 3e,f).

### 2.2 Crystal Structure Transformation of MnHCF in ARZIB

**Figure 4:**
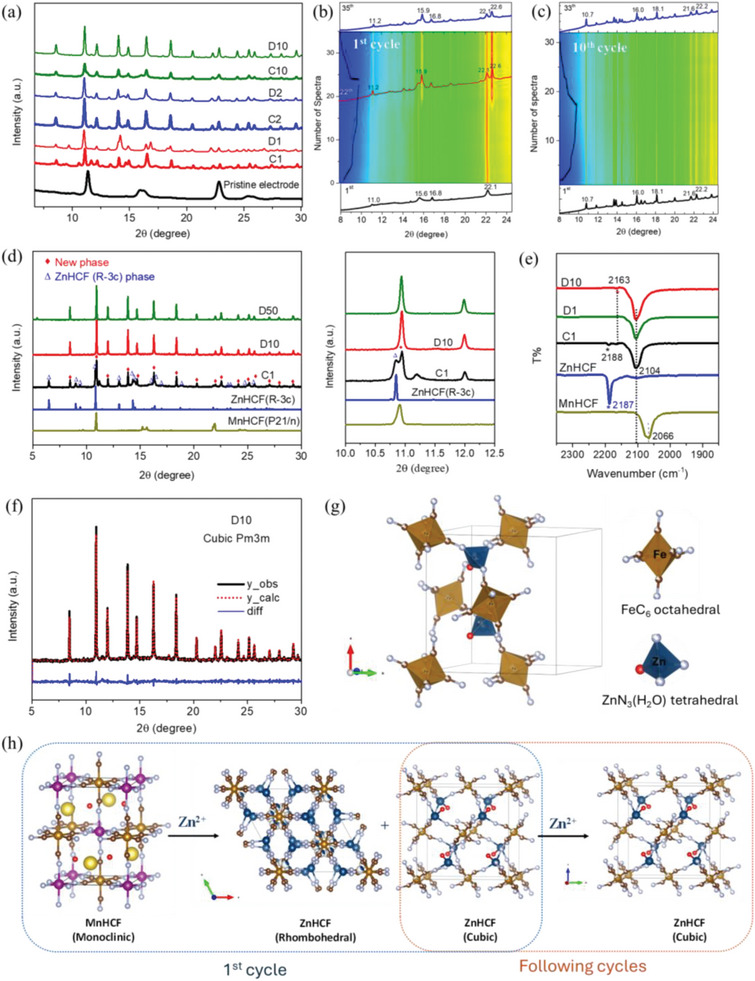
a) Ex situ synchrotron XRD data of MnHCF electrode at different charge/discharge states (pristine, C1, D1, C2, D2, C10, and D10 electrodes). Contour map of MnHCF operando synchrotron XRD data at b) 1st cycle; c) 10th cycle. d) The high resolution XRD data of C1, D10, and D50 electrodes. e) Ex situ FTIR spectra of MnHCF C1, D1, and D10 electrodes, as well as the powder MnHCF and ZnHCF spectra. f) Pawley refinement of D10 XRD data with cubic P m3m Bravais lattice. g) The atomic packing within the unit cell for Zn_2_[Fe (CN)_6_], while the Zn atom is tetrahedrally coordinated to three N ends and one water molecule. h) Schematic illustration of the crystal structure transformation of MnHCF during the first and the following cycles.

To identify the phase and crystal structure evolution of MnHCF during the insertion of Zn^2+^, ex situ and operando XRD data were collected. As reported,^[^ ^13^, ^14^, ^15^ ^]^ the phase transformation process of MnHCF in Zn^2+^‐containing aqueous electrolyte occurs mainly in the initial cycles, and here the same holds true, as shown in **Figure** **4**a. The original MnHCF monoclinic reflection peaks almost disappeared, and several new peaks were generated after the first charge. An identical XRD pattern was observed for the 2nd and 10th cycle electrodes, which confirms that the structural changes mainly happened during the first cycling process. To better understand the transformation process of MnHCF during the initial cycle, an operando XRD experiments was performed, and some peak changes are obviously visible from the contour map (Figure 4b,c). During the 1st cycle, first the electrode was charged to 1.9 V, which corresponds to the extraction of Na^+^ from the structure, the oxidation of Fe and Mn will happen at around 1.57 and 1.80 V,^[^ ^33^, ^34^ ^]^ respectively. Some new XRD peaks at around 11.2°, 15.9°, and 22.6°, appeared at the final stage of the charging process, which is related to the Mn oxidation process (>1.8 V). These experimental findings suggest that the structural transformation started during the oxidation process of the Mn. At the end of the 1st charge, the newly formed peaks are coexisting with the previous ones, but with less intensity at 11.2° and 15.9°. During the discharge process, those new peaks became dominant. The XRD pattern became quite stable by 9 cycles, no apparent changes were observed in the 10th cycle operando XRD data (Figure 4c). To get more precise information about the structure transformation, we collected the XRD data of C1, D10, and D50 electrodes by using a capillary geometry in high spin mode, as shown in Figure 4d. The data exhibited higher resolution, compare to the data collected from fixed pellet, which is required for further refining analysis. A mixture of two‐phases, i.e., the rhombohedral ZnHCF (R −3c) and a newly formed phase were clearly observed for C1 electrode. For D10 and D50 electrodes, the ZnHCF (R −3c) phase disappeared, while newly formed phase became the only existing stable phase that participates in the subsequent electrochemical reaction. This result is not consistent with the previous reports,^[^ ^13^, ^14^, ^15^ ^]^ which generally believed that the rhombohedral ZnHCF phase was the final stable phase.

The FT‐IR data (Figure 4e; and Figure S5, Supporting Information) further support the XRD result. The two tiny peaks at 2188 and 2163 cm^−1^ observed for C1 electrode, which can be attributed to the typical ν(CN) vibration peak of rhombohedral ZnFe^III^HCF (R −3c) and cubic ZnFe^III^HCF (F m‐3m) phases.^[^ ^35^, ^36^ ^]^ All the cycled electrodes (C1, D1, and D10) exhibit the same peaks of ν(CN) vibration at around 2104 cm^−1^, δ(FeCN) bending at 592.5 cm^−1^, and ν(FeC) vibration at 492.4 cm^−1^, according to the report of Rodriguez‐Hernandez et al.,^[^ ^35^ ^]^ a mixture phase of ZnFe^II^HCF (R −3c and F m‐3m phase) may be present in all cycled electrodes. The relatively high frequency for the ν(CN) band in a ferrocyanide suggests a high possibility of tetrahedral coordination for the zinc atom.^[^ ^37^ ^]^

To gain a complete understanding of the newly formed phase, a refinement analysis was conducted, based on the high‐quality D10 and D50 XRD data. The refinement work begins without considering the Mn in the framework, because the newly formed MnO_2_ phase only exhibits a local octahedral configuration, without a long‐range ordered crystal structure. After peak indexing and Pawley refinement, we found a cubic P m3m (a = 12.093 Å) Bravais lattice, that matched very well with the D10 XRD data, as shown in Figure 4f. In view of the information derived from the IR data, a structure based on a FeC_6_ octahedra and ZnN_3_(H_2_O) tetrahedra linked by cyanide bridging was built. In this structure the Zn atom coordinated to three N ends plus a water molecule to form a deformed tetrahedron (Figure 4g). The same configuration has been reported for the rhombohedral phase of ZnHCF,^[^ ^37^ ^]^ but is the first time to be found in a cubic ZnHCF phase. The details on the refinement parameters, the refined atomic positions, occupation, and thermal factors are available in Figure S6 and Table S2 (Supporting Information). The phase transformation of MnHCF during cycling can be illustrated as Figure 4f: during the 1st cycling process, the monoclinic MnHCF transformed to rhombohedral and cubic ZnHCF phase, while during the following cycles, the cubic ZnHCF phase became the only stable phase.

**Figure 5:**
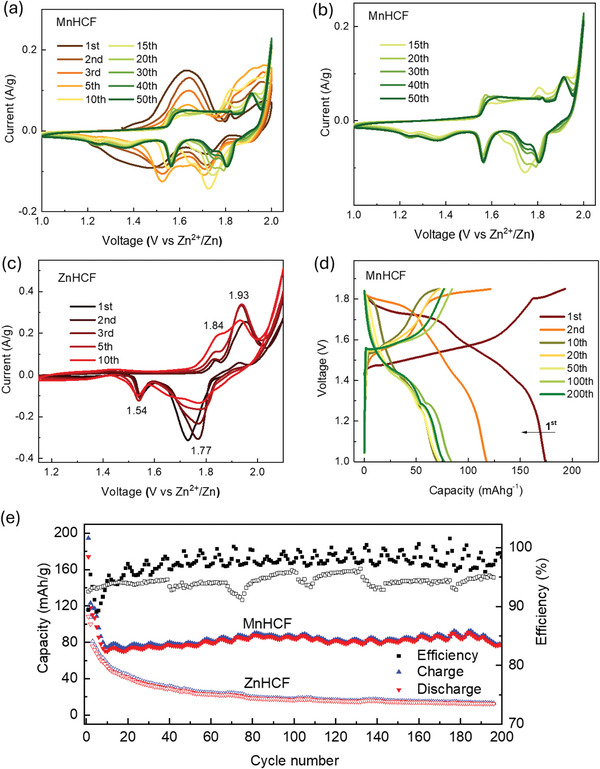
a) CV data of MnHCF ARZIB in 3 m ZnSO_4_ electrolyte at scan rate of 0.2 mV s^−1^ for 50 cycles. b) CV curves of ≈15th–50th cycles. c) CV data of ZnHCF ARZIB in 3 m ZnSO_4_ electrolyte. d) Galvanostatic charge/discharge curves of MnHCF ARZIB at C/5 in 3 m ZnSO_4_ electrolyte. e) Cycling stability of MnHCF and ZnHCF at 20 mA g^−1^ in 3 m ZnSO_4_ electrolyte at room temperature.

The phase and composition change also reflected in the electrochemical data. In principle, if the electrode material simply transforms from MnHCF to ZnHCF (cubic or rhombohedral phase), the electrochemical performance of the new ZnHCF compound is expected to be like the pure ZnHCF (R −3c) material. However, based on the CV peaks in **Figure** **5**a,b, we observed that the CV peaks of MnHCF completely changed during the first 10 cycles, and kept quite stable pattern only from 15th to 50th cycles. The anodic peaks at around 1.57, 1.82, and 1.91 V, as well as the cathodic ones at around 1.24, 1.37, 1.55, 1.70, and 1.80 V kept their positions, but changed their intensities. Compared with the CV data of pure ZnHCF electrode (Figure 5c), some similarities can be found between MnHCF cycled (10th−50th cycles) and the ZnHCF initial (1st–5th cycles) CV data, for example, the anodic peaks at around 1.82 and 1.91 V in MnHCF sample versus at around 1.84 and 1.93 V in ZnHCF sample, and the cathodic peaks at around 1.55, 1.70, and 1.80 V in MnHCF sample versus at around 1.54 and 1.77 V in ZnHCF sample, which can be attributed to the redox peaks of newly formed ZnHCF inside MnHCF structure during cycling. The remaining signals: the anodic peak at 1.57 V and two cathodic ones at around 1.24, 1.37 V for MnHCF electrode, were found very close to the redox peaks of MnO_2_.^[^ ^17^, ^19^, ^20^, ^38^, ^39^, ^40^ ^]^ The Galvanostatic charge/discharge curves of MnHCF ARZIB exhibited a high initial discharge capacity at around 174 mAh g^−1^, while the capacity was fading very fast during the first 10 cycles.). The discharge plateau above 1.7 V (vs Zn^2+^/Zn) gradually disappeared, which should be associated with redox of Mn inside MnHCF framework. Base on the discharge potential, the specific capacity contribution below 1.4 V increased from 15.5% at 1st cycle to 43.6% at 10th cycle, remained relatively stable until 200th cycle (Figure S7, Supporting Information), which might be attributed to the new formed MnO_2_ phase. Compared to the pure ZnHCF (R −3c) powder, MnHCF in ARZIB exhibited a much higher specific capacity and cycling stability. In other words, the newly formed compound, based on cubic ZnHCF framework and the amorphous MnO_2_, demonstrates much better electrochemical performance than the pure ZnHCF sample, and is highly promising for further Zn^2+^ storage.

## 3 Conclusion

Combining multiple X‐ray techniques, the local and long‐range structural changes of MnHCF electrode in aqueous ZnSO_4_ electrolytes were comprehensively studied. Based on the XAS and XRF results, the local configuration modifications of all the metals sites, Fe, Zn, and Mn, were clearly displayed. During cycling, the pristine ‐Mn‐NC‐Fe‐framework was mostly transformed to ‐Zn‐NC‐Fe‐, and the local structural rearrangement mainly occurred on Mn site. The EXAFS fitting results confirm the cleavage of the Mn*─*N bond, with the formation of a Mn*─*O bond, in an octahedral environment, and MnO_2_ as a main manganese‐ “survival” component in the cathode was proposed. Although no MnO_2_ reflection peaks were observed from the XRD data, the typical redox peaks of MnO_2_ were observed from the electrochemical tests. Furthermore, the XRD results confirmed that the phase transformation of MnHCF took place exclusively during the 1st cycle, and a mixture of rhombohedral and cubic ZnHCF phases were formed during the first charge process. Later, the new cubic ZnHCF phase became the only stable phase participating in the subsequent cycling and exhibiting high cycling stability. Based on the refinement of powder XRD data, a cubic ZnHCF unit cell, with Fe atom octahedrally coordinated to C ends and Zn atom tetrahedrally coordinated to three N ends plus a water molecule, was built and reported for the first time. The signature and evolution of the electrochemical data is fully consistent with the interpretation of the proposed structural and electronic modification upon the Zn ion insertion, providing a full picture of the structure–property–performance relationship in this class of materials. This research not only provides a deeper understanding of the working principle of MnHCF in ARZIB, but also offers strategies and methodologies for the development and interpretation of other battery systems in the future research.

## 4 Experimental Section

### Synthesis of MnHCF and ZnHCF

The synthesis of MnHCF was reported.^[^ ^9^ ^]^ Using a simple and reproducible coprecipitation method, manganese sulfate monohydrate (MnSO_4_·H_2_O, 0.1 m, 100 mL) solution, and sodium ferrocyanide decahydrate (Na_4_Fe (CN)_6_·10H_2_O, 0.1 m, 100 mL) were simultaneously added dropwise to an aqueous solution of sodium sulfate (Na_2_SO_4_, 0.1 m, 100 mL) by using a peristaltic pump at a rate of 4 mL min^−1^. Both the reagents and the reaction batch were kept under N_2_ atmosphere at a constant temperature (40 ± 2 °C) using a water bath. The obtained solution was aged for 5 days, ensuring the complete decantation. Then the precipitate was collected via centrifugation at 4000 rpm, washed three times with distilled water and dried at 60 °C for 48 h. The chemical formulation of MnHCF is Na_1.41_ Mn [Fe(CN)_6_]_0.89_. The ZnHCF powder was synthesized as ref. [24] with chemical formula Zn[Fe(CN)_6_]_0.862_.

### Electrode Preparation and Electrochemical Tests

The electrochemical properties of the obtained material were collected by using three electrode split test cell, in which the active material was utilized as a working electrode, while zinc sheet as a reference and a counter electrode. The working electrode was prepared by mixing the active material (70%), super C65 (25%), and PTFE (5%), and grinding until homogenous thin solid disc was got. Then a puncher was used to get 8 mm (diameter) pellets with a mass density of around ≈5–10 mg cm ^−2^.

Cyclic voltammetry (CV) was performed by means of CH Instruments model 660. The CV test was conducted in the potential range of 1.0–2.0 V versus Zn^2+^/Zn in 3 m ZnSO_4_ aqueous solution.

Galvanostatic cycling with potential limit (GCPL) was conducted in 1.0 < E <1.9 V versus Zn^2+^/Zn potential window at 20 mA g^−1^. Cycling started after a rest time (5 h) at OCP condition with a positive imposed current. All the ex situ electrodes were collected at 20 mA g^−1^, the abbreviation C1 means the first charge, and D1—the first discharge. The same naming method is used for C2, D2, C10, D10, and D50.

### Characterization

Infrared (IR) spectra were measured using a Bruker Alpha FT‐IR spectrometer in ATR (Attenuated Total Reflectance) mode at a spectral range of 4000–400 cm^−1^.

Powder X‐ray diffraction (PXRD) data were recorded using a monochromatic X‐ray beam (wavelength of 1 Å) at the MCX beamline in Elettra synchrotron Trieste (Italy).^[^ ^41^ ^]^ The diffraction patterns of powder samples were collected in a capillary geometry, setting the spinner at 3000 rpm with a high‐count rate fast scintillator detector. Electrode samples were tested in transition mode with a marCCD detector. Several diffraction patterns were collected consecutively in the range between 5° < 2θ < 50°, with steps of 0.01° and an acquisition time of 1 s step^−1^. The crystal structure was refined using GSAS‐II software.^[^ ^42^ ^]^

XRF measurements were carried out in the vacuum chamber available at the XRF beamline in Elettra synchrotron Trieste.^[^ ^43^, ^44^ ^]^ The energy of the primary X‐ray beam was selected using a Si (111) double crystal monochromator. The sample was placed at 45° with respect to the incident beam and to the silicon drift detector (SDD, Bruker Nano GmbH, XFlash 5030). The SDD has 131 eV energy resolution at Mn Kα, a 30 mm^2 ^active area, and it is equipped with an ultrathin polymer window. The distance between the sample and the detector was set to 15 mm. For the acquisition of the elemental maps, the excitation energy was fixed to 10.0 keV, and the beam footprint on the sample surface was 140 × 100 µm^2^ (H × V). The spectra were analyzed using PyMca.^[^ ^45^ ^]^ The spectra fitting is based on the fundamental parameters. The XANES spectra on the hot spots, where the spots are interested in, were collected at the K‐edge of Zn and Mn in fluorescence geometry using the same experimental set‐up. The data were analyzed using the Athena software.^[^ ^46^ ^]^

XAS experiments were conducted at Elettra Synchrotron Trieste (Italy), at XAFS beamline.^[^ ^47^ ^]^ Data were recorded at the Fe, Mn, and Zn K‐edge in transmission mode using ionization chambers filled with a mixture of Ar, N_2_, and He to have 20%, 70%, and 95% of absorption in the I_0_, I_1_, and I_2_ chambers, respectively. An internal reference of iron, manganese, and zinc foils were used for the energy calibration in each scan. This allowed a continuous monitoring of the energy during the consecutive scans. The white beam was monochromatized using a fixed exit monochromator equipped with a pair of Si (111) crystals. Mn, Fe, and Zn K‐edge spectra were collected from 6345 to 7100 eV, from 6920 to 8350 eV, and from 9467 to 10 897 eV, respectively.

### Statistical Analysis

XAS data pretreatment was conducted using the Athena program,^[^ ^46^ ^]^ which includes the XANES normalization procedure. The pre‐edge background was removed by subtraction of a linear function extrapolated from the pre‐edge region, and the raw spectra were normalized to the unity by extrapolation of the atomic background evaluated using a polynomial function. XANES spectra were normalized to an edge jump of unity. A prior removal of the background absorption was done by subtraction of a linear function extrapolated from the pre‐edge region. The EXAFS analysis was performed using the GNXAS package,^[^ ^48^ ^]^ which is based on multiple scattering (MS) theory. The method is based on the decomposition of the EXAFS signals into a sum of several contributions, the n‐body terms. It allows the direct comparison of the raw experimental data with a model theoretical signal. The procedure avoids any filtering of the data and allows a statistical analysis of the results. EXAFS data analysis is performed by minimizing a χ^2^‐like residual function that compares the theoretical signal to the experimental one. The errors for the structural parameter’ determination are displayed in Table S1 (Supporting Information).

## Conflict of Interest

The authors declare no conflict of interest.

## Supporting information

Supporting Information

## Data Availability

The data that support the findings of this study are available from the corresponding author upon reasonable request.
