# Supplementary material for: Structural Evolution of Manganese Prussian Blue Analogue in Aqueous ZnSO4 Electrolyte
Source: Small. 2024 Aug 6;20(51):2404584. doi: 10.1002/smll.202404584 (PMC11657057; doi:10.1002/smll.202404584)
Supplement: Supplementary file 1 — Supporting Information [file SMLL-20-2404584-s001.docx]

Supporting information

**Structural Evolution of Manganese Prussian Blue Analogue in Aqueous ZnSO_4_ Electrolyte**

*Min Li, Mariam Maisuradze, Angelo Mullaliu, Ilaria Carlomagno, Giuliana Aquilanti, Jasper Rikkert Plaisier, Marco Giorgetti ^*^*

Figure S1. (a-c) XRF spectra of C1, D1 and C10 electrodes. (d) the molar ration of Mn/Fe and Zn/Fe.


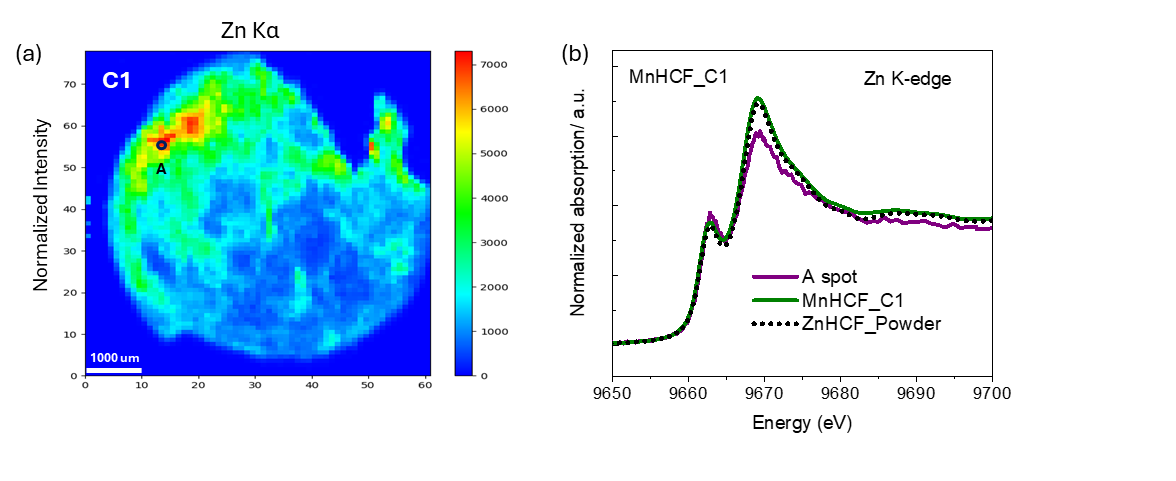


Figure S2. (a) XRF elemental map of Zn Kα peak for C1 electrode. (b) XANES spectra of A spot, compared to the Zn k-edge of C1 electrode and ZnHCF powder, which were collected at XAFS beamline at Elettra.


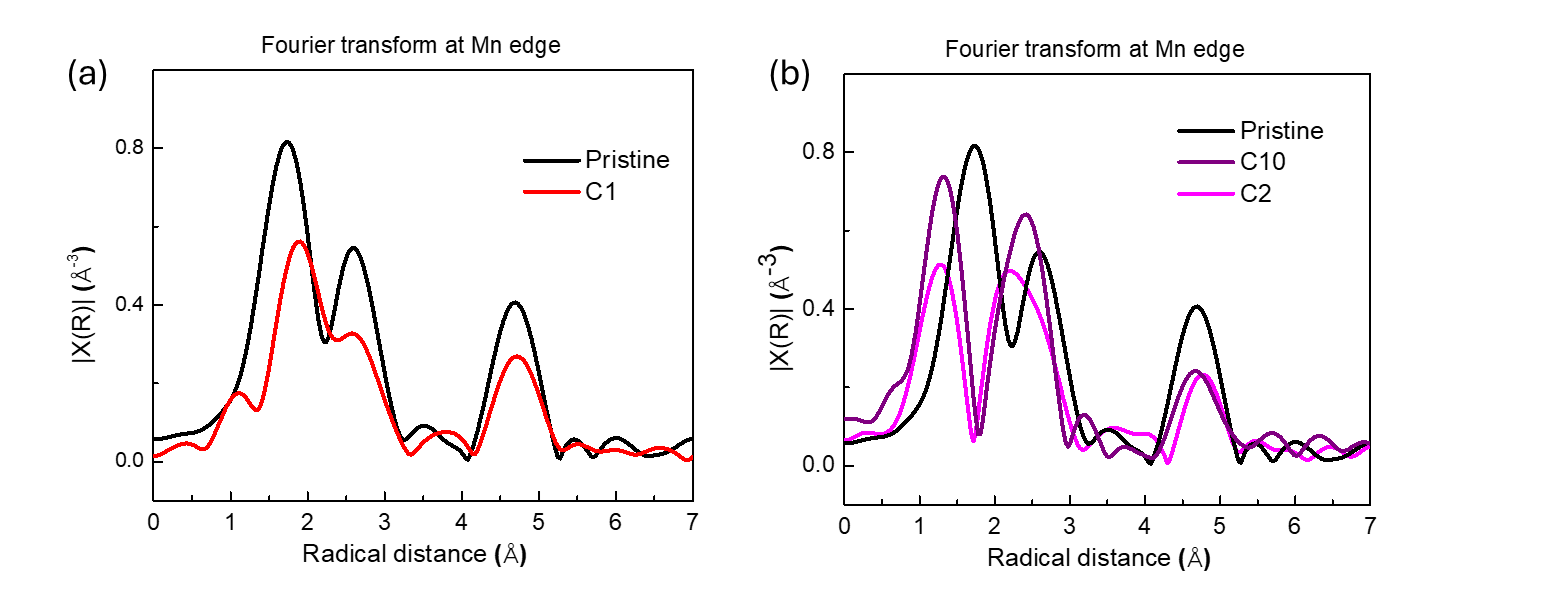


Figure S3. Fourier transform (FT) of Mn k^2^-weighted EXAFS signal for (a) C1 electrode. (b) C2 and C10 electrodes. Compared with the pristine electrode.


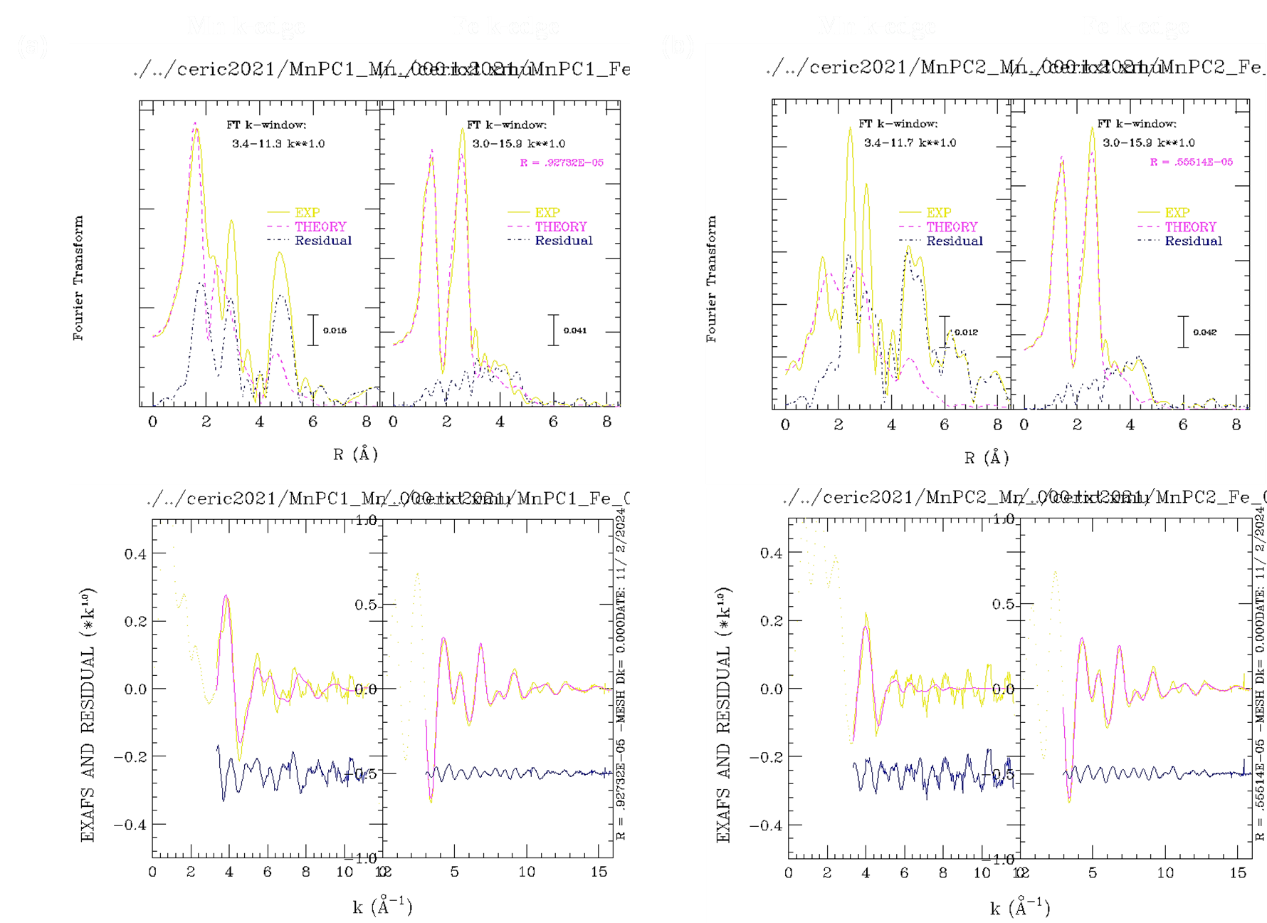


Figure S4. EXAFS signals and comparison of the best fit total signal with the experimental data of the Fe k-edge and Mn k-edge for (a) C1 electrode and (b) C2 electrode.

Table S1. EXAFS fitting parameters considering one (MnHCF) or two (MnHCF and MnO_2_) models. Errors in parameters have been obtained by two-dimensional section of the parameter space (contour plots).

|  | **One structural model (MnHCF)** | | | **Two structural models**  **(MnHCF and MnO_2_)** |
| --- | --- | --- | --- | --- |
|  | **Pristine** | **1^st^ charged**  **C1*** | **10^th^ charged**  **C10*** | **10^th^ charged**  **C10** |
| Fe-C (N) / Å  σ^2^ Fe-C / Å^2^ | 1.878(4)  0.0015(3) | 1.888  0.0018 | 1.878  0.0017 | 1.876(3)  0.0018(3) |
| C≡N / Å  σ ^2^ C≡N / Å^2^ | 1.180(4)  0.010(2) | 1.170  0.010 | 1.172  0.008 | 1.172(3)  0.005(1) |
| Mn- N / Å  σ^2^ Mn / Å^2^ | 2.183(6)  0.008(2) | 2.117  0.008 | 2.172  0.028 | 2.10(2)  0.012(3) |
| θ Fe-C-N / deg | 175 FIX | 175 FIX | 175 FIX | 180 FIX |
| θ Mn-N-C / deg | 175 FIX | 175 FIX | 175 FIX | 180 FIX |
| σ ^2^ Fe-C-N / deg^2^ | 45(20) | 44 | 36 | 45(6) |
| σ ^2^ Mn-N-C / deg^2^ | 5(3) | 22 | 54 | 4(3) |
| Mn-O / Å  σ^2^ Mn-O / Å^2^ | - | - | - | 1.816(5)  0.018(5) |
| Mn- - Mn / Å  σ^2^ Mn- -Mn / Å^2^ | - | - | - | 2.94(3)  0.013(4) |
| % MnMCF model  % MnO_2_ model | - | - | - | 20(5)  62(8) |
| E_0_ Mn | 6542.1(5) | 6540 | 6548 | 6548(1) |
| E_0_ Fe | 7117.7(5) | 7118 | 7118 | 7117.6(5) |
| S_0_^2^ Mn | 0.65(5) | 0.65 | 0.73 | 0.66(6) |
| S_0_^2^ Fe | 0.73(2) | 0.80 | 0.78 | 0.80(7) |
| χ^2^-like residual /(10^-6^) | 6.63 | 9.27 | 9.07 | 3.62 |


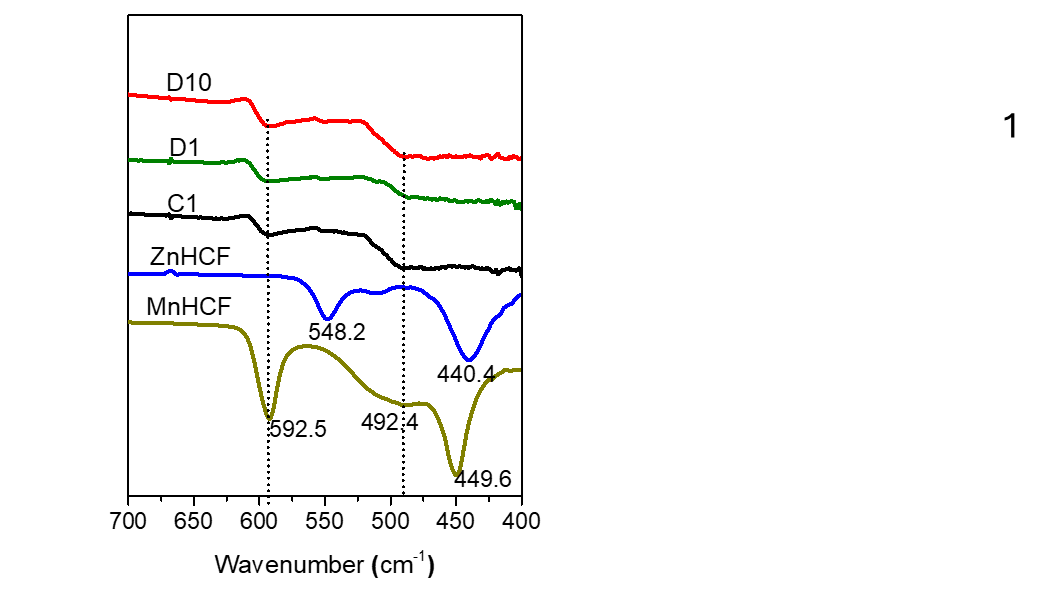


Figure S5. ex-situ FTIR spectra of MnHCF C1, D1 and D10 electrodes, as well as powder MnHCF and ZnHCF spectra, at low wavenumber.

Figure S6. (a) Rietveld refinement of D10 XRD data with cubic ZnHCF phase. (b-c) Crystal structure cubic ZnHCF and Mn added ZnHCF structure.

Table S2. Atomic position, occupation, and thermal factors of cubic ZnHCF phase.

| **Space group: P m3m** | | | | | |
| --- | --- | --- | --- | --- | --- |
| **Atom** | **x** | **y** | **z** | **frac** | **U**$\boldsymbol{iso}$ |
| **Fe** | **0** | **0** | **0** | **0.72** | **0.004** |
| **Zn** | **0.173** | **0.673** | **0.826** | **0.70** | **0.02** |
| **C** | **0.070** | **0.615** | **0.423** | **0.72** | **0.08** |
| **N** | **0.073** | **0.736** | **0.423** | **0.72** | **0.08** |
| **Mn** | **0.119** | **0.331** | **0.088** | **0.30** | **0.28** |
| **O** | **0.274** | **0.274** | **0.274** | **1.00** | **0.08** |


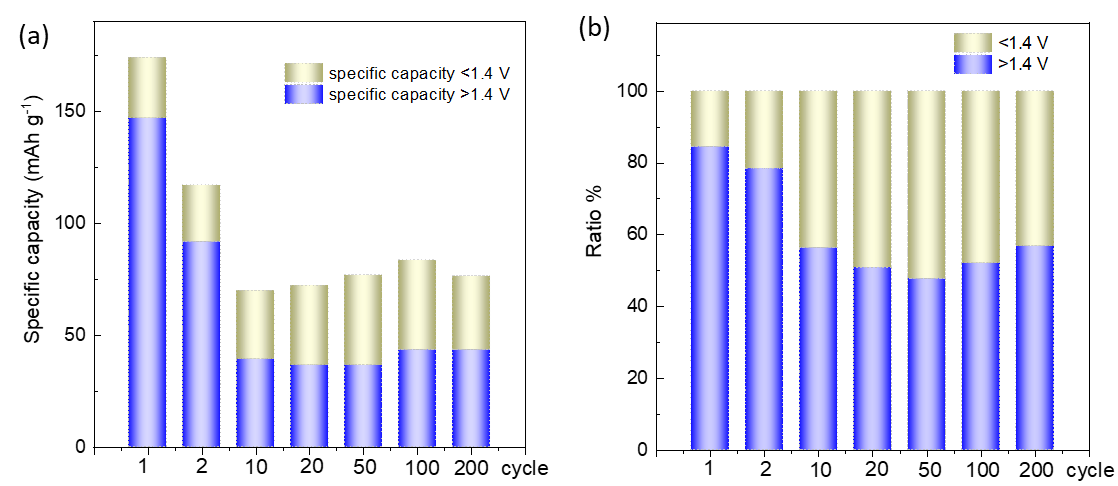


Figure S7 (a) Specific capacity contribution from potential below 1.4 V and above 1.4 V; (b) the specific capacity contribution ratio of different potential range.
